# 

**Published:** 1900-07

**Authors:** 


*Fifty-Fifth Year.*

VOL. LV.
WHOLE NUMBER,
DCXLIV.

JULY, 1900.

New Series.
VOL. XXXIX.
No. 12.

### BUFFALO MEDICAL JOURNAL

**Established 1845 by AUSTIN FLINT, M. D.**

**EDITOR:**

WILLIAM WARREN POTTER, M. D.

**ASSISTANT EDITORS:**

WILLIAM C. KRAUSS, M. D. NELSON W. WILSON, M. D.

**ASSOCIATE EDITORS:**

JAMES WRIGHT PUTNAM, M. D.
ERNEST WENDE, M. D.
JOHN PARMENTER, M. D.
ALVIN A. HUBBELL, M. D.
JOHN A. MILLER, PH. D.
MAUD J. FRYE, M. D.

LIBRARY
Indexed

LIBRARY
SURGEON GENERAL'S OFFICE
JUL.-5--1900

SCRIPTA FERUNT ANNOS

EUROPEAN AGENCY, J. B. BAILLIERE & FILS, 19 Rue Hautefeuille, Paris.
*Subscription, if paid in advance, $2.00; otherwise, $2.50. Foreign subscription, $2.50*
Copyright 1900, by WILLIAM WARREN POTTER, M. D.
Entered at the Post Office at Buffalo, N. Y., as second-class mail matter.

A. T. BROWN PRINTING HOUSE, BUFFALO, N. Y.

**Table of Contents on Page V.**

